# Beyond Traditional Risk Calculators: The Expanding Role of Coronary Artery Calcium Scoring in Preventive Cardiology

**DOI:** 10.7759/cureus.93500

**Published:** 2025-09-29

**Authors:** Sai Praneeth Chaparala, Navin Sampathkumar, Sreeleela Jonnadula, Aishwarya Chand, Dipanjan Chowdhury

**Affiliations:** 1 Internal Medicine, Gayatri Vidya Parishad Institute of Health Care and Medical Technology, Visakhapatnam, IND; 2 Medicine, Kasturba Medical College, Manipal, Manipal, IND; 3 Cardiology, Katuri Medical College and Hospital, Guntur, IND; 4 Internal Medicine, Ruby Hall Clinic, Pune, IND; 5 Pulmonary Medicine, Institute of Post Graduate Medical Education and Research and Seth Sukhlal Karnani Memorial Hospital, Kolkata, IND

**Keywords:** artificial intelligence, aspirin, atherosclerosis, cac scoring, cardiovascular risk, coronary artery calcium, preventive cardiology, primary prevention, risk stratification, statin therapy

## Abstract

Coronary artery calcium (CAC) scoring has emerged as an increasingly recognized and guideline-endorsed tool in cardiovascular risk stratification, particularly for asymptomatic individuals at intermediate risk. This narrative review synthesizes data from 109 peer-reviewed studies (2000-2025) to evaluate the current clinical utility, limitations, and future potential of CAC scoring in the primary prevention of atherosclerotic cardiovascular disease (ASCVD). Robust evidence demonstrates that CAC offers superior prognostic value compared to traditional risk estimators such as the Framingham Risk Score and pooled cohort equations, effectively guiding decisions on statin and aspirin therapy. A CAC score of 0 reliably predicts very low event rates and holds potential for de-escalating preventive therapy, yet remains underused. Technological advances, including artificial intelligence (AI)-driven CAC quantification from non-gated chest CT scans, present scalable and low-cost opportunities for broader implementation. However, significant barriers persist, including inconsistent guideline integration, radiation concerns, economic limitations, and the absence of large randomized controlled trials (RCTs) validating outcome benefits. Future research must establish population-specific CAC thresholds, validate AI tools in real-world settings, and generate robust RCT data to confirm clinical impact. Integrating CAC scoring into routine preventive care could redefine personalized cardiovascular risk assessment and close critical gaps in equitable ASCVD prevention.

## Introduction and background

Overview of coronary artery calcium scoring and its role in primary prevention

Coronary artery calcium scoring (CACS) is a non-invasive CT technique that helps in the diagnosis and treatment of subclinical calcified atherosclerosis, which progresses to atherosclerotic cardiovascular disease (ASCVD). ASCVD is a major cause of sudden cardiac death in myocardial infarction [1]. CACS is the product of the total calcified area of plaque (in mm2) and the peak calcium density factor. CACS determines the degree of coronary artery disease (CAD) in each atherosclerotic lesion [2,3]. The biological basis and natural course of atherosclerosis show a positive correlation between calcium density and age. Lesion vulnerability and the risk of atherosclerotic cardiovascular disease have an inverse relationship with calcium density in population-based cohorts when age and plaque area are considered. Independent of conventional risk variables, a calcium density >1,000 HU (1,000 plaque) is linked to a reduced chance of acute coronary syndrome [3]. CACS had a stronger tendency for risk prediction among susceptible patients than the traditional Atherosclerotic Cardiovascular Disease (ASCVD) score, the Multi-Ethnic Study of Atherosclerosis (MESA), and the Rotterdam Study (RS) [4,5]. In addition, among patients with borderline and intermediate risk who were initially assessed using pooled cohort equations, coronary artery calcium (CAC) had additive prognostic significance [6,7]. When used in conjunction with nuclear myocardial perfusion imaging (MPI, elliptically encased region), CACS can improve the risk assessment of CAD and be a potentially advantageous application [8].

**Figure 1:**
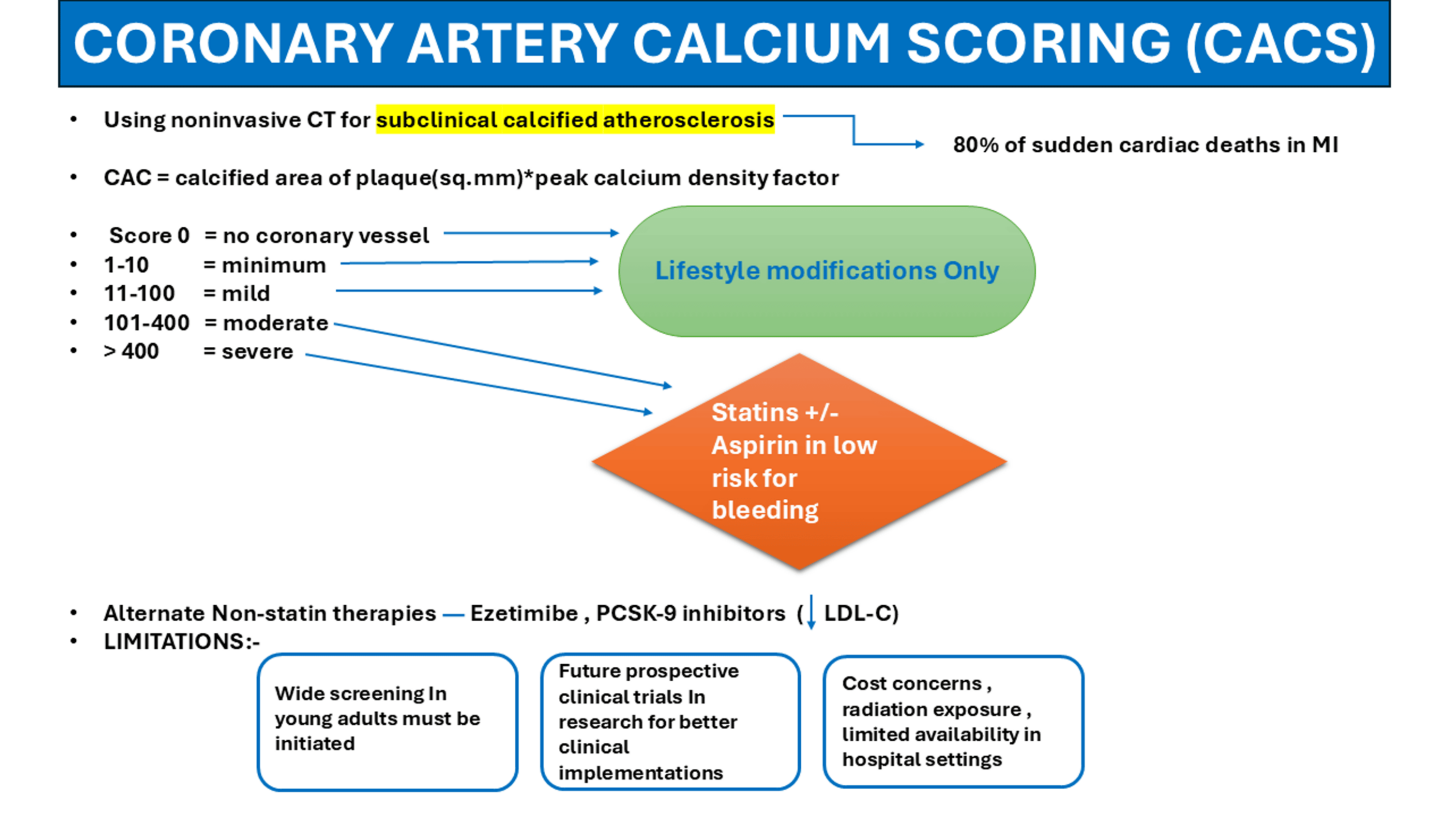
Overview of CACS for diagnosing and treating subclinical calcified atherosclerosis. Approach to treatment based on scoring: 0 = no coronary disease, 1-10 = minimum, 11-100 = mild, 101-400 = moderate, > 400 = severe. A score of 0-100 indicates LSM only. A score of >100 indicates statin ± aspirin use in patients with a low risk of bleeding. Alternate non-statin therapies to lower LDL-C include ezetimibe and PSCK-9 inhibitors. CACS = coronary artery calcium Scoring; LSM = lifestyle modifications; PCSK-9 = proprotein convertase subtilisin kexin type 9; LDL-C = low-density lipoprotein cholesterol; MI = myocardial infarction The figure has been created by the authors. No previously published material has been reproduced; therefore, no permissions or citations were required.

Following CAC score estimation, each coronary vessel is assigned an absolute CAC score based on the maximal HU. These scores can be linked to a visual score in which 0 denotes no coronary vessel, 1-10 minimum, 11-100 mild, 101-400 moderate, and >400 severe. Visual scores are subjective assessments of CAC derived from the visual examination of images that are not ECG-gated [9,10]. A CAC score of 0 has a very low annualized mortality rate, delaying the initiation of statin therapy, except for familial hypercholesterolemia and diabetes, where screening is required [11,12]. For high CAC scores (>100), statin therapy is recommended, as the 10-year risk of major atherosclerotic cardiovascular events (MACE), such as myocardial infarction (MI) and stroke, exceeds >7.5%. Moreover, it has been demonstrated that International Classification of Diseases and Related Health Problems, 9th Revision codes for MI and stroke have ≥90% positive predictive value (PPV) for capturing confirmed clinical MI and stroke incidents [13-15]. For patients with CAC scores >100 and no risk of bleeding, aspirin therapy is advised by the National Lipid Association (NLA) for primary prevention [13,14]. Guidelines for alternative lipid-lowering treatments, such as ezetimibe and proprotein convertase subtilisin kexin type 9 inhibitors, have been provided by the 2022 American College of Cardiology (ACC) Expert Consensus Decision Pathway on the Role of Non-Statin Therapies for Low-Density Lipoprotein Cholesterol (LDL-C) Lowering. For primary prevention, these guidelines for the first time contain specific LDL-C treatment goals based on the CAC score [13,16]. Similarly, even when traditional CVD risk factors exist, a CAC score of 0 has been related to a reduced risk of coronary heart disease (CHD). In patients at borderline to intermediate risk (5-20% 10-year ASCVD risk) where statin medication decisions are unclear, the 2018 ACC/American Heart Association (AHA) guidelines on blood cholesterol treatment suggested using CAC scoring [12]. The approach to treatment using CACS is summarized in Figure 1.

Coronary artery calcium vs. traditional risk calculators: which provides better risk stratification

One of the most critical challenges in clinical cardiology is the early identification of individuals at a heightened risk for CAD events, such as MI or sudden cardiac death. Traditional risk stratification models, such as the Framingham Risk Score (FRS) in the United States and HeartScore in Europe, primarily rely on demographic and clinical characteristics (age, sex, diabetes status, systolic blood pressure, total cholesterol, high-density lipoprotein cholesterol (HDL-C), and smoking history) to estimate the 10-year risk of cardiovascular events in individuals without established CAD [17]. Over the years, several similar risk calculators have been developed from large cohort studies, as summarized in Table 1, which outlines the widely used traditional risk calculators for ASCVD [18].

**Table 1:** Various types of traditional risk factor calculators. FHS: Framingham Heart Study; ASSIGN: a Scottish cardiovascular risk estimation system; QRISK2: QRISK cardiovascular risk algorithm version 2; SCORE: Systematic Coronary Risk Evaluation; PROCAM: Prospective Cardiovascular Münster Study; CVD: cardiovascular disease; CHD: coronary heart disease; BP: blood pressure; HDL-C: high-density lipoprotein cholesterol; HbA1c: glycated hemoglobin A1c; BMI: body mass index; NA: not applicable; ✓: variable included in the model

	FHS (1991) [19]	FHS (2008) [20]	ASSIGN [21]	QRISK2 [22]	SCORE [23]	Reynolds [24]	PROCAM [25]
Valid age range (years)	30–74	30–74	30–74	30–84	40–65	45–80	20–75
Cohort men (%)	46%	47%	49%	50%	57%	40%	68%
Follow-up (years)	12	12	10–21	7.1	13.2	10.2	11.7
Location	US	US	Scotland	England	Europe	US	Germany
Outcome type	CVD	CVD	CVD	CVD	Fatal CVD	CVD	CHD
Model type	Parametric	Cox	Cox	Cox	Weibull	Cox	Weibull
Gender	NA	✓	✓	✓	✓	✓	✓
Smoking	✓	✓	✓	✓	✓	✓	✓
Systolic BP	✓	✓	✓	✓	✓	✓	✓
Total cholesterol	NA	✓	✓	NA	✓	✓	✓
HDL-C	NA	✓	✓	NA	NA	✓	✓
Total/HDL	✓	NA	NA	✓	✓	NA	NA
HbA1c	NA	NA	NA	NA	NA	✓	NA
Diabetes	✓	✓	✓	✓	NA	✓	✓
Ethnicity	NA	NA	NA	✓	NA	NA	NA
BMI	NA	NA	NA	✓	NA	NA	NA
Family history	NA	NA	✓	✓	NA	✓	NA
BP treatment	NA	✓	NA	✓	NA	NA	NA

However, these models have limitations, particularly in terms of individual risk discrimination. For example, they do not capture the subclinical atherosclerotic burden or consider biomarkers or imaging findings that may offer more personalized risk stratification. In this context, the CAC scan, a non-contrast chest CT acquired during a breath-hold, has emerged as a promising tool for improving cardiovascular risk prediction. By measuring calcified plaques throughout the epicardial coronary arteries and using a threshold of 130 HU over a contiguous area of at least 3 pixels or 1 mm², the CAC scan identifies radiopaque lesions that signify subclinical atherosclerosis [26].

A growing body of literature supports the superior prognostic performance of CACS over that of traditional models. A 2023 study by Khan et al. compared CACS with polygenic risk scoring in two large cohorts from the United States and the Netherlands. CACS significantly improved risk discrimination when added to pooled cohort equations (PCEs), with a net reclassification improvement (NRI) of 0.09 (95% CI = 0.06 to 0.28), whereas polygenic risk scores showed minimal reclassification benefit (NRI 0.04; 95% CI = -0.05 to 0.10) [4]. The prognostic value of CACS has been demonstrated in long-term cohort studies. In a five-year follow-up of 10,377 asymptomatic individuals, Shaw et al. (2003) showed that CACS was a stronger independent predictor of all-cause mortality than traditional risk factors were. The addition of CAC scores to a multivariable Cox model improved the concordance index from 0.72 to 0.78 (p < 0.001) and enhanced the outcome classification (AUC = 0.73 vs. 0.67, p < 0.001) [27].

The clinical utility of CAC also extends to the guidance of preventive pharmacotherapy. In a 9-year observational study involving 13,644 patients without baseline ASCVD, Mitchell et al. (2018) found that statin therapy was associated with a significantly reduced risk of major adverse cardiovascular events (MACE) in patients with CAC > 0 (adjusted subhazard ratio: 0.76; 95% CI = 0.60-0.95; p = 0.015), but no benefit was observed in those with a CAC score of 0 (subhazard ratio = 1.00; 95% CI: 0.79-1.27; p = 0.99). The number needed to treat (NNT) to prevent one MACE ranged from 100 (CAC 1-100) to 12 (CAC >100) [15]. These findings highlight how CACS complements and improves upon traditional models, such as FRS, which fail to account for interindividual variation in plaque burden. Importantly, biomarkers such as highly sensitive C-reactive protein (hsCRP) and CAC can improve risk stratification, especially in patients deemed low- or intermediate-risk by the FRS. For instance, Nasir et al. showed that individuals with a family history of early-onset CAD were more likely to have CAC >0, regardless of their FRS classification [28].

Moreover, CAC’s predictive utility appears to be robust across ethnicities, including White, Black, Hispanic, and Chinese Americans. A doubling of the CAC score was associated with a 1.5-fold increased risk of major coronary events [29]. Its predictive value also extends beyond coronary events to include stroke and atrial fibrillation, as evidenced by multiple studies. In certain contexts, CAC may outperform hsCRP, especially in predicting non-fatal MIs and broader cardiovascular outcomes, as demonstrated by Park et al. in a non-diabetic population [30]. Despite their strengths, traditional calculators such as the FRS, ASSIGN, QRISK2, and Reynolds remain limited. These models assess the current health status but do not reflect prior exposure to risk factors or an individual’s biological susceptibility to atherosclerosis. Consequently, they may overmedicate older patients while under-identifying young individuals at genuine risk. CAC scans offer a critical advantage by visualizing the subclinical plaque burden, particularly in asymptomatic and young adults, promoting primary prevention and treatment optimization [31-33].

However, it is important to acknowledge the key limitations of CAC. One major drawback is its inability to detect non-calcified plaques, which are more prevalent in younger individuals and may represent early-stage high-risk lesions [22]. Moreover, there is a lack of randomized controlled trials (RCTs) proving the non-inferiority or superiority of the CAC score over other risk scores. For instance, the St. Francis Heart Study did not show statistically significant improvements in ASCVD outcomes with statin use in patients with CAC >80th percentile [34]. The feasibility and funding of large-scale RCTs remain major barriers, as evidenced by the unfunded status of the Greenland et al. proposal [35]. Looking ahead, non-randomized data and ongoing trials, such as ROBINSCA [36] and the 10-year DANCAVAS follow-up [37], are expected to shed further light on whether CAC-guided screening improves long-term cardiovascular prognosis.

However, controversy persists. In a 2022 systematic review and meta-analysis by Bell et al., six cohort studies were analyzed to assess the incremental benefit of CAC when added to traditional CVD risk calculators. The findings indicated modest gains; most participants reclassified as intermediate- or high-risk based on CAC scores remained event-free (85-96%) over 5-10 years. These modest benefits may not justify radiation exposure, incidental findings, and out-of-pocket costs associated with CAC scanning in lower-risk populations [5]. While CACS is a powerful tool, particularly for stratifying risk in low-to-intermediate risk individuals and guiding personalized prevention strategies, it is not without limitations. Future insights from long-term population-based trials are essential to define the clinical thresholds and cost-effectiveness of CAC-guided screening across diverse patient populations.

## Review

Methodology

This narrative review was conducted using a structured search strategy. We systematically searched PubMed, Scopus, and Google Scholar for studies published between 2000 and 2025 and historical foundational articles, using the following keywords: “coronary artery calcium,” “CAC scoring,” “risk stratification,” “primary prevention,” “statin therapy,” “aspirin,” and “artificial intelligence in CAC.” Inclusion criteria comprised (1) peer-reviewed human studies focused on the role of CAC in primary prevention, (2) English-language publications, and (3) studies reporting cardiovascular outcomes, prognostic performance, or implementation strategies. We included 109 studies, spanning large cohort investigations (e.g., MESA), guideline statements (ACC/AHA, ESC, TSOC, SCCT), meta-analyses, and cost-effectiveness analyses. Exclusion criteria were case reports, conference abstracts, non-peer-reviewed commentaries, and studies limited exclusively to secondary prevention or diagnostic coronary CT angiography. As this was a narrative rather than a systematic review, we acknowledge that the design carries a risk of selection bias, and results should be interpreted with this limitation in mind. The heterogeneity in study designs, populations, and outcome measures limits the harmonization of guidelines and the broad clinical adoption of CACS-based strategies. To address this, shared decision-making models can incorporate CACS results by individualizing preventive strategies, communicating absolute and relative risk, and integrating patient preferences across diverse healthcare settings. While AI-driven automation enhances workflow efficiency, its long-term impact on clinical outcomes remains uncertain and requires validation through longitudinal studies. Open questions regarding optimal repeat testing intervals, cumulative radiation exposure, and implications for cost-effectiveness and patient safety are discussed, emphasizing the need for standardized follow-up protocols. Furthermore, ongoing trials such as ROBINSCA, DANCAVAS, and CAC-PREVENTABLE may influence not only guideline recommendations but also reimbursement policies and broader clinical practice. Finally, we acknowledge that many existing studies underrepresent younger adults, women, and non-European ethnicities, limiting the generalizability of current findings and highlighting the importance of inclusive research for equitable implementation.

Should coronary artery calcium scoring guide statin and aspirin therapy in primary prevention?

Cardiovascular risk prediction plays a pivotal role in guiding preventive strategies, such as statin and aspirin use. While clinical risk calculators are widely used in practice, their predictive power is limited, particularly for individuals at borderline or intermediate risk, where treatment decisions are most ambiguous. This uncertainty can lead to either overtreatment, exposing patients to unnecessary adverse effects, or undertreatment, missing opportunities to prevent major cardiovascular events [38]. CACS has emerged as a potent imaging biomarker that enhances risk stratification beyond that of traditional risk factors. It provides direct visualization of the calcified atherosclerotic burden, allowing for a more accurate estimation of risk and guiding treatment with greater precision [29]. Individuals with CAC scores of 0 or <100 consistently exhibit very low cardiovascular event rates, even in the presence of multiple risk factors [39]. In contrast, elevated CAC scores, particularly >100 or >30, are associated with a significantly higher risk and may justify the early initiation of statins and, in select cases, low-dose aspirin [39,40]. In fact, Individuals with undetectable CAC often experience a reclassification from high-risk to low-risk status, leading to more tailored treatment decisions [40]. Recognizing its value, the ACC/AHA and other professional societies now recommend CACS as a useful decision-making tool when the benefit of treatment remains uncertain [11]. Current guidelines for aspirin use in CVD use different CAC thresholds, leading to confusion for clinicians. These variations are due to differences in risk modeling approaches, event rate assumptions, and health system priorities, rather than disagreement about CAC’s prognostic power. Aspirin recommendations are extrapolated from broader ASCVD risk reduction trials, with CAC used as a modifier of baseline risk. The ACC/AHA guidelines cautiously support aspirin use in select patients with elevated CAC, while the U.S. Preventive Services Task Force (USPSTF) guidelines discourage routine aspirin use due to bleeding concerns.

The prognostic validity of CAC is supported by several large-scale studies, particularly in individuals aged 45-75 years [41-43]. For example, Vliegenthart et al. (2005) analyzed 1,795 asymptomatic participants in the Rotterdam Study and found a graded increase in ASCVD risk with higher CAC scores (adjusted hazard ratios = 3.1 for 101-400, 4.6 for 401-1000, and 8.3 for >1,000 compared to <100) [44]. Similarly, a pooled analysis of U.S. cohorts (Framingham, MESA, and CHS) by Yano et al. (2017) revealed that CAC was a stronger predictor of ASCVD and stroke than chronological age alone [45]. Compared to other serum biomarkers, CACS consistently shows a superior predictive value for future events [46].

Beyond its utility in guiding statin initiation, CACS also aids in aspirin decision-making for primary prevention. In the MESA study, Miedema et al. (2014) evaluated 4,229 non-diabetic participants stratified by the FRS and CAC levels [47]. They found that low CAC (0-99) was associated with net harm from aspirin use due to the bleeding risk. A high CAC (>100) yielded a net benefit, with a favorable balance between ASCVD prevention and bleeding risk. The estimated NNT for a five-year benefit ranged from 92 to 173, depending on the risk tier, whereas the number needed to harm (NNH) due to major bleeding was consistently around 442. This highlights the importance of CACS as a critical filter to avoid indiscriminate aspirin use [47]. Multiple international societies now endorse the CAC as a valuable tool for refining ASCVD risk assessment and treatment personalization.

American College of Cardiology/American Heart Association 2019 Guidelines [48]

CACS is recommended for adults aged 40-75 years with intermediate risk (7.5%-20%) when statin decisions are uncertain. A CAC score of 0 may support deferring statin therapy. A score of 1-99 suggests a possible benefit, especially for patients over 55 years of age. A score of ≥100 strongly supports statin initiation. See Table 2 for full treatment recommendations [48].

**Table 2:** Full treatment recommendations. CAC = coronary artery calcium; ASCVD = atherosclerotic cardiovascular disease

CAC score	Statin recommendation	Aspirin recommendation	Notes
0	May withhold statins in intermediate-risk adults	Not recommended	Consider other risk enhancers (e.g., family history, smoking, diabetes)
1–99	Consider statins, especially if age >55 years or other risk enhancers	Not routinely recommended	Supports shared decision-making
>100 or >75th percentile	Recommend statins regardless of age	May consider in adults 40–70 years with low bleeding risk	Strong indicator of subclinical atherosclerosis

Taiwan Society of Cardiology 2024 Guidelines [49]

Taiwan Society of Cardiology (TSOC) guidelines advocate CAC for borderline (3-7%) or intermediate (7-10%) 10-year CAD risk groups. It emphasizes the use of CAC as a tool to up- or down-classify individuals and guide therapy intensity (Table 3).

**Table 3:** TSOC stratification and intervention thresholds. CAC = coronary artery calcium; TSOC = Taiwan Society of Cardiology

CAC score	Statin recommendation	Aspirin recommendation	Notes
0	May withhold statins	Not recommended	Reassess in 5 years unless high risk
1–99	Lifestyle changes; consider statins depending on percentile/risk	Not routinely recommended	Strong emphasis on percentiles and risk assessment
>100	Recommend initiating statin therapy	May consider low-dose aspirin in low-bleeding-risk adults	Based on stronger correlation with cardiovascular events
>400	Recommend high-intensity statins	Recommend aspirin if bleeding risk is low	Considered high risk by imaging

U.S. Preventive Services Task Force 2022 Guidelines [50]

The guidelines advise individualized aspirin therapy for adults aged 40-59 years with ≥10% ASCVD risk. Aspirin is not recommended for those aged ≥60 years due to the risk of bleeding. CAC can inform aspirin decisions by identifying patients who are more likely to benefit from aspirin therapy. See Table 4 for USPSTF-aligned CAC risk stratification.

**Table 4:** USPSTF-aligned CAC risk stratification. USPSTF = United States Preventive Services Task Force; CAC = coronary artery calcium; LDL-C = low-density lipoprotein cholesterol; ASCVD = atherosclerotic cardiovascular disease

CAC score	Statin recommendation	Aspirin recommendation	Notes
0	Consider deferring statins (unless high-risk features like smoking, diabetes, or family history)	Not recommended	No detectable plaque
1–99	Favor statins if risk factors persist (e.g., LDL >160 mg/dL, family history)	Not routinely recommended	Mild plaque burden
>100 or >75th percentile	Strongly recommend statins regardless of age + aggressive lifestyle changes	Recommended if no bleeding risk	High plaque burden
>300 or >90 percentile	Statins recommended	Aspirin is recommended if the bleeding risk is low	High-risk plaque burden

Society of Cardiovascular Computed Tomography 2021 Guidelines [51]

According to the Society of Cardiovascular Computed Tomography (SCCT), CACS is recommended for asymptomatic individuals aged 40-75 years with a 5%-20% 10-year risk or <5% with a strong family history. They recommend CAC ≥100 as a clear trigger for statin therapy (Table 5).

**Table 5:** SCCT-aligned guidance based on LDL-C and other factors. SCCT = Society of Cardiovascular Computed Tomography; CAC = coronary artery calcium; LDL-C = low-density lipoprotein cholesterol; ASCVD = atherosclerotic cardiovascular disease

CAC score	Statin recommendation	Aspirin recommendation	Notes
0	Defer statin therapy in adults aged 40–75 with LDL-C of 70–189 mg/dL, no diabetes, smoking, or family history of premature ASCVD	Not recommended	Avoid statins in adults aged 76–80 when the decision is uncertain
1–99	Consider statin therapy, especially in patients over 55 years of age	Not routinely recommended	Mild atherosclerosis. Repeat CAC scoring in 3–5 years if results may influence treatment decisions
>100 or >75th percentile	Initiate statin therapy	Consider aspirin therapy if no contraindications	Moderate risk for atherosclerosis
>300 or >90 percentile	Initiate high-intensity statin therapy. Consider additional LDL-C lowering therapies to achieve >50% reduction and LDL-C <70 mg/dL	Aspirin is recommended if bleeding risk is low	High risk for atherosclerosis

**Figure 2:**
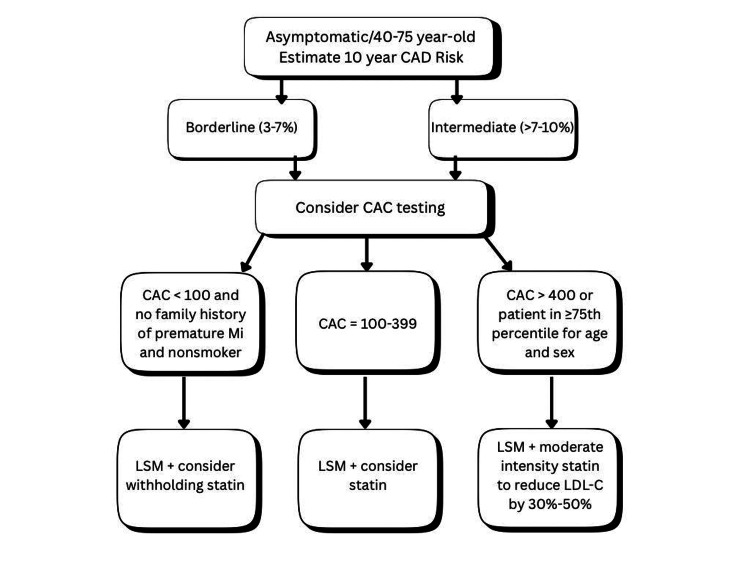
Broader role of coronary artery calcium in risk assessment and therapeutic decision-making. The figure was created by the authors. No previously published material has been reproduced; therefore, no permissions or citations were required. CAC = coronary artery calcium; CAD = coronary artery disease; MI = myocardial infarction; LDL-C = low-density lipoprotein cholesterol; LSM = lifestyle modifications

Figure 2 illustrates the broader role of CAC in risk assessment and therapeutic decision-making, emphasizing its integration with lifestyle modifications, LDL-C management, and aspirin therapy.

CACS improves the benefit-to-harm ratio of preventive therapies by allowing targeted interventions. In individuals with CAC ≥100, the NNT for aspirin or statin therapy was substantially lower, reflecting high treatment efficiency [52]. In contrast, those with a CAC score of 0 often derive minimal cardiovascular benefit and face a disproportionate risk of bleeding from aspirin. Avoiding unnecessary treatment in these patients prevents adverse events and contributes to healthcare cost savings.

Health Economics Perspective

In Australia, the CAC scanning costs ~AUD 198. For CAC ≥100, the incremental cost-effectiveness ratio (ICER) was AUD 33,108 per quality-adjusted life year (QALY) gained, which was considered cost-effective. For low CAC scores, the ICER was AUD 53,028 per QALY, which was still within the acceptable thresholds [53]. In the United States, CAC scans cost USD 100-200. Economic models show that CAC-guided statin therapy yields ICERs of USD 20,000-70,000 per QALY, depending on population risk and adherence [54]. Cost-effectiveness is particularly evident in the following areas. Older men, particularly >60 years, those with high LDL-C, individuals with a family history of premature CAD, and low socioeconomic populations, where CAC-guided therapy may boost adherence. Moreover, CAC-guided strategies reduce first-time CAD and CVD events more than traditional models and have been shown to improve long-term adherence to therapy compared to traditional models [53,54]. CACS is a clinically validated, guideline-endorsed, and economically justified tool for primary prevention. It facilitates personalized risk stratification, minimizes overtreatment, enhances treatment precision, and supports better resource allocation in both public and private healthcare systems.

Research gaps in coronary artery calcium utilization and implementation

**Figure 3:**
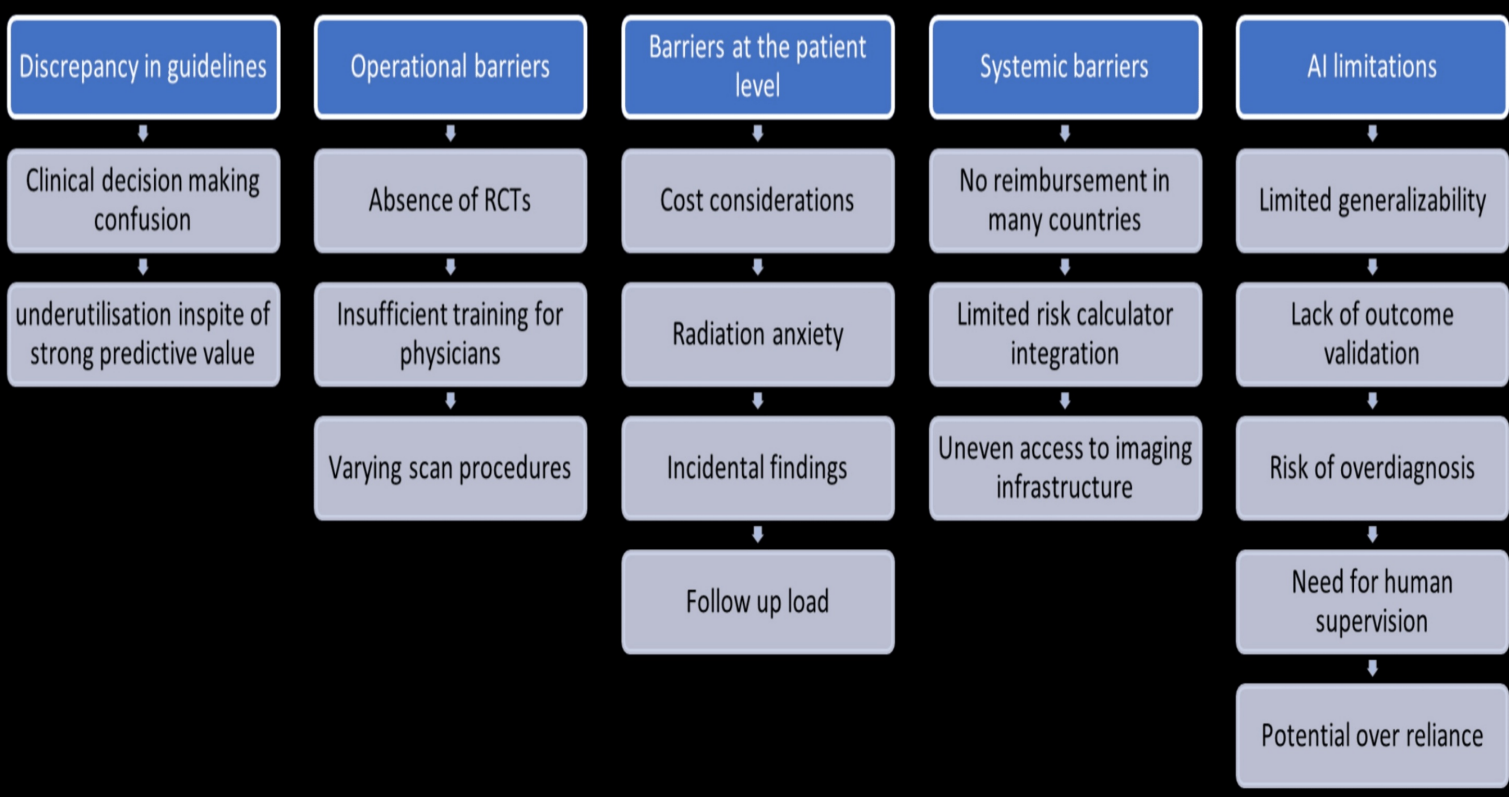
Research gaps in the use of coronary artery calcium scoring. The figure was created by the authors. No previously published material has been reproduced; therefore, no permissions or citations were required.

Although CACS has shown significant promise in enhancing the prediction of cardiovascular risk, there are still important research and implementation gaps. CACS has the potential to improve statin medication recommendations, which currently result from the gap between the European Society of Cardiology (ESC) and AHA/ACC guidelines. Although standard guidelines prescribe lifetime statin therapy for individuals with a modest atherosclerotic burden, there is evidence that CACS can identify those who may safely skip this treatment. However, it may also support therapy for people at moderate risk who are disregarded by current procedures [55] (Figure 3).

Figure 3 summarizes key barriers across the following five domains: (1) guideline discrepancies causing clinical confusion and underuse; (2) operational issues such as lack of RCTs, physician training, and scan variability; (3) patient-level concerns including cost, radiation anxiety, and follow-up burden; (4) systemic barriers such as limited reimbursement, risk calculator integration, and infrastructure access; and (5) AI limitations involving generalizability, validation gaps, overdiagnosis risk, and overreliance.

However, there is a lack of agreement on when and how to apply CAC across groups, contributing to its inadequate integration into guideline-based decision-making. Although CAC has a higher prediction accuracy than traditional algorithms, its application in clinical practice is still not optimal. For example, the AHA/ACC recommendations recommend statins for many people, particularly older adults, but CAC may assist in avoiding overtreatment by identifying people who are actually low-risk individuals. However, certain high-risk patients may not receive the recommended amount of treatment under the ESC guidelines, which the CAC can help close. Finally, a crucial research and implementation gap is highlighted by the absence of consistent recommendations and the underutilization of CAC in therapy customization [55]. The clinical consequences of under-detection of non-calcified plaque, particularly in symptomatic patients, are outlined, and circumstances where additional imaging may be warranted. The role of calcium density in modifying cardiovascular risk was elaborated, highlighting why uncertainties persist despite growing evidence. Limitations of applying CACS in very elderly adults were addressed, including its relevance beyond statin initiation, and the manuscript now discusses how missed opportunities in young adults with MI <50 years can inform trial design by identifying high-risk subgroups for preventive strategies. The ethical and economic implications of opportunistic AI-based screening, including risks of overdiagnosis and incidental findings, were incorporated, alongside clarification of the populations and comorbidities affected by the lack of RCT evidence.

Compared with conventional models, CACS offers enhanced risk classification in primary prevention. However, depending on whether ESC or AHA/ACC recommendations are applied, different clinical judgements based on CAC grading are made. This emphasizes the lack of evidence-based, standardized methods for integrating CAC into cardiovascular risk management. Despite its high predictive power, CAC is not widely used in clinical practice. Limited RCTs, inadequate integration of guidelines, and a lack of knowledge on its function across various age, sex, and ethnic groups are contributing problems [55]. According to Nasir et al., approximately 41% of patients recommended to take statins under the 2013 ACC/AHA guidelines could be reclassified with a CAC score of 0, potentially saving them from needless therapy [56]. However, owing to issues such as radiation exposure, cost, access to imaging, and physician confidence, CAC remains underutilized in practice. Further data are required to standardize the use of CAC, verify its cost-effectiveness, and establish its usefulness in different therapeutic contexts [56,57].

Importantly, even among people with conventional risk factors, a CAC score of 0 has been associated with extremely low 10-year incidence rates, indicating that many people might safely forego statin therapy and instead concentrate on changing their lifestyles. For example, after a 10-year period, only 4.9% of individuals with a CAC score of 0 who were administered statins had cardiac incidents. The incident rate was only 1.5% in the intermediate-risk group (10-year ASCVD risk of 5%-7.5%), even though 57% of them had CAC=0. Therefore, CAC testing is essential for improving statin selection, especially in patients with intermediate risk [56].

Notwithstanding its advantages, CAC testing has drawbacks, including a $100 price tag, accidental results (such as non-cardiac nodules), and a modest but noticeable radiation dose (~0.89 mSv). However, its broad acceptance has been constrained by these elements and practical issues, including workflow disruption and downstream testing. Furthermore, additional research is required to validate the potential benefits of CAC in various populations, even though it may enhance medication compliance and lifestyle adherence [56]. Using calculators, the 2013 ACC/AHA cholesterol guidelines shifted the emphasis from LDL-C objectives to the overall ASCVD risk. Despite its novelty, this strategy has sparked discussions. Because the CAC rating has a higher predictive potential, it provides a supplementary tool. The usefulness of CAC in individualized therapy was further supported by Nasir et al. [58], who discovered that people with high CAC scores but no traditional risk factors had higher all-cause mortality than those with numerous risk factors but no CAC [57-60]. The debate on universal versus selective CAC screening is influenced by cost-effectiveness, population-level outcomes, and ethical concerns. Selective approaches are preferred due to resource constraints and risk heterogeneity. Cost-effectiveness analyses have been limited, with uncertainties about subgroup implications in younger women, ethnic minorities, and lower-risk populations. Evidence on long-term adherence and behavioral modification after CAC disclosure is limited, and evidence-based protocols are needed. Barriers to AI implementation include interoperability, regulatory approval, and cost.

According to studies by Mirbolouk et al (2019) and Miedema et al. (2014), CAC enhances risk stratification in populations such as smokers and younger adults, where traditional models frequently fall short. While standard models based on demographic and clinical data, such as blood pressure, smoking status, diabetes, cholesterol, or age, may misclassify these individuals, CAC makes it possible to visualize and measure the burden of coronary plaque directly [61,62]. In addition to cardiovascular results, quality-of-life factors are considered when determining the worth of CAC. The hardship of taking pills, adverse drug reactions, and low-risk radiation-induced cancer have all been taken into account by researchers [63-65]. Despite the modest amount of radiation, it nonetheless plays a role, especially in high-dose or recurrent imaging. Furthermore, in 4%-8% of instances, CAC scans may reveal incidental non-cardiac abnormalities, such as lung nodules, that require follow-up. The findings raise the possibility of increased expenses and patient worry, even if the majority are not clinically significant [66-68].

The narrative review by Abdelrahman et al. highlighted that automated detection on non-gated chest CT is made possible by AI, expanding access without the need for further imaging or expense. It increases uniformity through standardized grading and boosts efficiency by lowering the burden on radiologists. While more validation and integration into standard workflows are still crucial, these developments promote a wider, more scalable application of CAC in healthcare [69]. According to the findings, AI-based CACS has progressed from early rule-based models (sensitivity ~74%) to sophisticated deep learning systems with ICC ≥0.9 and sensitivity >90%. These models perform well across CT modalities, save analysis time by approximately 60%, support opportunistic screening in preventive care, and exhibit a significant correlation with manual assessment (Pearson r = ≈0.96-0.98) [69]. AI-based CACS employing attenuation correction CT (CT-CAC) scans provides an economical and radiation-free technique for opportunistic cardiovascular risk assessment during regular imaging. It enhances effectiveness, lowers variability, and has a strong correlation with myocardial perfusion. However, its low independent predictive value highlights the necessity for additional research and clinical integration [70].

By using AI and machine learning for automation, Ihdayhid et al. (2022) and Winkelmann et al. (2022) overcame several important research and implementation gaps in the application of CACS. Both studies address a major operational barrier, time and resource intensity, by showing that completely automated CAC scoring systems may substantially shorten analysis time to 13 seconds and 5.9 seconds, respectively, compared to traditional manual or semi-automated approaches. This effectiveness makes it more feasible to incorporate CACS into regular clinical procedures and extensive screening. Accuracy, repeatability, and standardization are crucial for wider clinical applications, and both models demonstrated outstanding agreement with experienced readers, with ICCs exceeding 0.90. By introducing artery-specific scoring, Winkelmann et al. further improved the clinical value and made it possible to analyze the disease load in greater depth. High classification accuracy for risk categories was also shown in both studies, assisting with specific treatment choices. When combined, these developments in automated CACS offer workable, scalable answers to important research gaps, such as inefficiencies, a lack of standardization, and restricted access, opening the door for a more widespread and successful application of CAC in cardiovascular risk estimation and preventive care [71,72].

Originally designed for lung cancer screening, automated CACS on low-dose CT (LDCT) scans allows for simultaneous cardiovascular risk assessment without the need for further radiation exposure or expense. AI makes it possible to assess CAC rapidly and precisely, enabling the early identification of coronary artery disease in those who do not exhibit any symptoms. This makes it easier to implement timely preventive measures, such as statin therapy or lifestyle modifications. This dual-purpose strategy closes important gaps in the prevention of CVD while improving efficiency and cost-effectiveness [73]. Cost-effectiveness analyses of CACS show that ICERs can be acceptable depending on country-specific thresholds and payer perspectives. However, indirect costs such as productivity loss and caregiver burden are underexplored. Evidence for improved medication adherence is mainly from observational cohorts, but modeled assumptions project greater adherence effects. Women and younger adults present distinct challenges, and repeated CAC scanning maintains long-term cost-effectiveness. Health system factors and probabilistic sensitivity analyses are crucial in economic evaluations, as expanding CAC testing could divert resources from alternative preventive interventions.

Future of coronary artery calcium screening: clinical implications and technological innovation

Coronary Artery Calcium Scoring in Preventive Cardiology

CACS is a well-validated, non-invasive imaging marker that directly reflects the coronary atherosclerotic burden and serves as a powerful predictor of future cardiovascular events. With the rapid advancements in AI and imaging technology, CACS has become a game-changing tool in the landscape of preventive cardiology. It enables personalized decision-making, particularly for lifestyle modification and statin therapy, in individuals at risk of ASCVD [74]. Typically performed via ECG-gated CT, CACS provides additional risk stratification, particularly for asymptomatic individuals at borderline or intermediate risk (5%-20% 10-year ASCVD risk) [75]. The 2019 ACC/AHA guidelines support CAC-guided decisions in these populations. A score of 0 often allows for safe deferral of statin therapy, whereas scores ≥100 strongly support statin initiation and more intensive preventive care [48]. However, the ideal scope of CAC use remains debatable. Although its predictive power is widely acknowledged, questions remain regarding whether CAC screening should be universally applied or used more selectively [76,77]. Some experts advocate for broader CAC screening in middle-aged, asymptomatic adults given its strong prognostic value, while others caution against overuse due to cost, limited insurance coverage, radiation exposure, and the potential for anxiety related to incidental findings, especially in individuals likely to have a score of 0 [77]. Cost-effectiveness analyses reflect this tension. In intermediate-risk men, CAC-guided prevention strategies have demonstrated favorable ICERs. However, in women and lower-risk groups, the ICERs can exceed $500,000 per QALY, suggesting limited clinical utility in these populations [78]. Furthermore, a CAC scan may cost roughly the same as a year of generic statin therapy, raising practical concerns regarding financial sustainability [79].

The emergence of AI has considerably expanded the possibilities for opportunistic CAC detection in asymptomatic patients. Deep learning algorithms, such as convolutional neural networks (CNNs) and U-Nets, have demonstrated high accuracy (91% sensitivity for non-zero CAC) and strong agreement with manual scoring (Spearman’s r = 0.90-0.92) [69]. These tools now enable the detection of CAC from non-gated chest CT scans, such as those used in routine lung cancer screening, without requiring dedicated cardiac imaging. This makes CACS more accessible, while minimizing radiation exposure and additional costs [69]. AI-based systems are also being developed to integrate CAC data directly into electronic health records, potentially streamlining real-time risk classification and clinical workflow [80].

Despite these advances, gaps persist in the real world. CAC remains underutilized in certain high-risk subgroups, such as adults under 45 years of age with familial hypercholesterolemia or autoimmune diseases [81]. Moreover, incidental CAC detected on non-cardiac CT scans is frequently underreported, with studies suggesting that only 44-59% of such findings are documented in radiology reports [82]. Additionally, while AI can enhance detection, its direct impact on clinical outcomes, such as treatment adherence, statin initiation, or reduction in adverse cardiovascular events, remains unclear [83]. The future of CAC-guided preventive care is being actively evaluated in several large RCTs. The ongoing CorCal (9,000 participants) and ROBINSCA (43,447 participants) trials are testing whether CAC-guided strategies result in improved long-term outcomes [84]. Similarly, the CAC PREVENTABLE trial is investigating whether the benefits of statins in older adults vary based on baseline CAC scores. The results of these trials, expected by 2027, could significantly shape the clinical guidelines and reimbursement policies. Existing evidence, especially from the Multi-Ethnic Study of Atherosclerosis (MESA), supports the clinical value of CAC. The MESA has shown that CACS enhances the performance of traditional risk models, such as the FRS, by both reducing overtreatment in low-risk patients (CAC = 0) and identifying higher-risk individuals previously misclassified (CAC >100). These dual benefits underscore CAC’s potential as a cornerstone of personalized prevention [85]. With AI-enhanced analysis and the opportunistic use of existing imaging, its clinical utility is expected to grow. However, widespread adoption will likely depend on forthcoming trial outcomes and continued integration into evidence-based guidelines.

Limitations, research gaps, and future directions of coronary artery calcium scoring

CACS has several important limitations across different populations. In young adults (<45 years), the low prevalence of CAC makes large-scale screening less cost-effective and reduces its ability to discriminate risk, as many individuals would need to be scanned to identify the small subgroup with high CAC [7,86-88]. Another limitation is the under-detection of non-calcified plaques, as CACS only detects calcified lesions and may miss early-stage or lipid-rich atherosclerosis. This creates the potential risk of false reassurance in symptomatic patients with a CAC score of 0 [2,89]. The use of CAC in symptomatic patients also remains debatable, as a score of 0 does not definitively exclude CAD in these individuals, highlighting the need for further trials [90-92]. Radiation exposure, although relatively low (~1 mSv, comparable to 120 days of background radiation or a single mammogram), still poses a concern, particularly for serial testing [93]. Moreover, most evidence supporting CACS arises from observational studies, with relatively few RCTs directly evaluating CAC-guided outcome modification [88,90]. Another challenge arises in very elderly patients (>75 years), where the high prevalence of CAC (>80%) limits its discriminatory power. However, it is noteworthy that a CAC score of 0 in older adults remains a strong negative risk marker, with a 98% five-year survival rate [94-96].

Several research gaps exist in CACS. Among young adults and patients with MI under the age of 50 years, many would not have qualified for statin therapy based on current guidelines before their event, emphasizing a missed opportunity for prevention [97-99]. Data from the CARDIA and CTA registries further indicate that approximately 10%-20% of asymptomatic adults under 45 years have CAC or detectable plaque, with CAC >100 being associated with a nearly fourfold increase in CHD mortality [100,101]. Designing RCTs in this population, however, is challenging due to the need for large sample sizes and long-term follow-up given the low event rates [102]. In older adults (>75 years), CACS may aid in personalizing statin use, but outcome data remain limited to intermediate timeframes of approximately 3.8 years [103]. Another unresolved question relates to the calcium density debate, as high CAC density may actually be protective, being linked to plaque stability and a reduced number of lipid cores [2,89,104].

AI applications in CACS introduce their own limitations. Most AI models have been developed on homogeneous or single-center datasets, which introduces training bias and limits generalizability across diverse populations [69,71]. Furthermore, there is a lack of long-term validation, as only a few models have been tested against meaningful clinical outcomes such as major adverse cardiovascular events (MACE) or medication adherence [71,72]. Infrastructure challenges also exist, with limited integration of AI-based CAC scoring into hospital picture archiving and communication systems, clinical workflows, and electronic health records [71,72]. Interpretative complexity remains another barrier, as artery-specific AI-derived scores currently lack standardized thresholds aligned with existing guidelines [72]. Finally, there is a risk of overdiagnosis, as opportunistic AI-based screening may identify incidental findings, leading to unnecessary follow-up investigations [73].

Looking ahead, several strategies may enhance the utility of CACS. In young adults, research should focus on targeting high-risk subgroups, such as those with a strong family history of premature CAD or multiple risk factors, for CAC-guided prevention trials. Serial coronary CTA could also be employed for tracking plaque progression in this population [102]. In older adults, embedding CAC substudies within large statin trials, such as the PREVENTable study, could help clarify its role in outcomes beyond cardiovascular events, including frailty, cognition, disability, and polypharmacy [103,105]. Future CACS models may be improved by incorporating extracoronary calcifications, such as aortic valve and mitral annulus calcification, to enhance risk prediction for stroke and CVD [106,107]. Cost efficiency and scalability could also be improved through fully automated CAC scoring, which may reduce overall costs by approximately 30% [104,108]. Finally, further development of AI-integrated approaches should prioritize robust clinical validation, alignment with guideline-based thresholds, and seamless integration with electronic health records to enable real-time decision-making support [69,71,72]. AI-based CAC quantification on non-gated chest CTs remains largely investigational. Several deep learning tools (e.g., CNN-based algorithms) have shown excellent correlation with manual scoring (ICC ≥0.9), but most are not yet FDA-approved for clinical use.

ACC/AHA (U.S.) recommends CACS for adults aged 40-75 years at borderline/intermediate risk (7.5%-20% 10-year ASCVD risk) when statin decisions are uncertain. A CAC score of 0 may allow statin deferral, while ≥100 strongly supports initiation. ESC (Europe) incorporates CAC into the SCORE2 framework, but thresholds are less explicitly defined, leading to variability in application. SCCT (U.S.) positions CAC more aggressively, with ≥100 as a clear statin trigger regardless of other risk factors. TSOC (Taiwan, 2024) recently adopted CAC for borderline (3%-7%) and intermediate (7%-10%) 10-year CAD risk groups, emphasizing percentiles. CSANZ (Australia/New Zealand)supports CAC in primary prevention, particularly for individuals with a family history of premature CAD, and highlights its role in adherence motivation. This lack of harmonization contributes to clinical uncertainty, physician hesitancy, and underuse of CAC in routine preventive cardiology practice.

## Conclusions

CACS represents a valuable adjunct to traditional risk assessment, offering superior prognostic precision and practical guidance in the allocation of preventive therapies such as statins and, in selected cases, aspirin. Its cost-effectiveness, capacity to refine decision-making in borderline and intermediate-risk groups, and potential integration with AI highlight its promise for broader clinical use. However, the evidence base is dominated by observational cohorts, with limited randomized trial validation, and disparities persist in guideline adoption, population-specific applicability, and health system implementation. Beyond cost and radiation exposure, CACS is limited by its inability to assess non-calcified plaque, and AI-based automation remains investigational. As a narrative review, our synthesis underscores these strengths and limitations without formal bias grading. Looking ahead, ongoing trials such as ROBINSCA, DANCAVAS, and PREVENTABLE are expected to clarify the clinical utility of CAC-guided interventions. Standardization of thresholds, validation across diverse populations, and careful integration of AI will be critical for realizing CAC’s long-term role in preventive cardiology.
